# Cloud-Based Behavioral Monitoring in Smart Homes

**DOI:** 10.3390/s18061951

**Published:** 2018-06-15

**Authors:** Niccolò Mora, Guido Matrella, Paolo Ciampolini

**Affiliations:** Dipartimento di Ingegneria e Architettura, Università degli Studi di Parma, 43124 Parma, Italy; guido.matrella@unipr.it (G.M.); paolo.ciampolini@unipr.it (P.C.)

**Keywords:** active and assisted living (AAL), smart home, behavioral analysis, deep learning, machine learning

## Abstract

Environmental sensors are exploited in smart homes for many purposes. Sensor data inherently carries behavioral information, possibly useful to infer wellness and health-related insights in an indirect fashion. In order to exploit such features, however, powerful analytics are needed to convert raw sensor output into meaningful and accessible knowledge. In this paper, a complete monitoring architecture is presented, including home sensors and cloud-based back-end services. Unsupervised techniques for behavioral data analysis are presented, including: (i) regression and outlier detection models (also used as feature extractors for more complex models); (ii) statistical hypothesis testing frameworks for detecting changes in sensor-detected activities; and (iii) a clustering process, leveraging deep learning techniques, for extracting complex, multivariate patterns from daily sensor data. Such methods are discussed and evaluated on real-life data, collected within several EU-funded projects. Overall, the presented methods may prove very useful to build effective monitoring services, suitable for practical exploitation in caregiving activities, complementing conventional telemedicine techniques.

## 1. Introduction

The demographics of the aging population are challenging social and economic equilibria of most countries [1]. The need of care increases with age, so that sustainability of the current social- and health-care systems is most severely endangered. A shift from a cure to prevention paradigm is recommended, which also implies focusing on prevention places (homes, living environments), in addition to treatment places (hospitals, sheltered houses). Supporting prevention and enabling home-based assistance is among the main goals of ambient assisted living (AAL, [2]) techniques.

Many technologies contribute to the AAL vision, predecessors of which can be found in home automation [3] and telemedicine technologies [4]. Many AAL systems rely on “smart-home” technologies, in which a wide variety of sensors are distributed in the home environment to infer behavioral patterns [5,6]. Such patterns can be exploited to activate home functions, e.g., automatic lighting, HVAC personalized setting, etc., aiming at comfort or energy-management purposes [7]. In a deeper perspective, sensors can be exploited to extract useful data for monitoring wellbeing and health; for instance, simple sensors can be exploited to track sleeping habits, which provide an expressive, yet indirect, health-related indicator. This may effectively complement classical telemedicine services. In fact, telemedicine provides information which is explicitly and univocally related to health, but inherently suffer from limited dimensionality (only a few clinical measurements are suitable for end-user self-assessment) and a lack of continuity (measurements are necessarily taken at discrete—i.e., not too frequent—time intervals). Moreover, telemedicine requires some skill to the end-user and strict following of the measurement schedule, which is possibly boring to the end-user himself and results in poor compliance, or even in making the approach unsuitable (e.g., in the case of cognitive impairment). Indirect behavioral monitoring, instead, can be carried out through home sensors with no burden on the end-user in an unobtrusive, continuative fashion. More (artificial) intelligence, however, is needed to infer meaningful knowledge from raw sensor data.

Recently, machine learning techniques have been applied to smart home data for a variety of monitoring/prediction tasks. For example, the CASAS system [8] was specifically designed to perform activity of daily living (ADL) recognition from a network of home sensors. The system exploits various classifiers, including support vector machines (SVM), hidden Markov models (HMM), and conditional random fields (CRF), with the authors reporting that best accuracy results were achieved with SVM. Other techniques were proposed for ADL classification: in [9], the authors describe a data mining approach to extract emerging recurrent patterns; an ontology-based classification, instead, is described in [10]. Finally, deep learning (DL) approaches have also been attempted for ADL prediction: in [11], the authors present a method based on Deep Belief Networks (DBN). As a further investigation step, the output of ADL recognition algorithms can be analyzed to assess the regularity of a user’s patterns. For example, in [12], a sensor data clustering approach is exploited to obtain insights into patterns and, at the same time, to detect deviant ones. On the other hand, [13] presents several methodologies to test for statistically significant differences in the detected patterns by comparing the current period to a reference one. Those methods included: (i) non-parametric tests that compare the probability distribution of epochs; (ii) permutation-based approaches; and (iii) a virtual classifier approach in which a classifier is trained to distinguish between patterns of the current window and the reference one. Furthermore, this approach was also adapted to physical activity trackers [14]. Interestingly, the authors in [15] show that the extracted daily living patterns and their relative changes can be good predictors of cognitive and mobility tests performed by clinicians.

In the literature, ADL discovery and classification typically rely on two factors: (i) a large number of sensors (especially PIR—passive infrared—motion detectors); and (ii) a significant corpus of user-annotated data. However, having complex installation and setups in real-life scenarios may be perceived as obtrusive by the system’s users. Furthermore, in most cases, thoroughly annotated data may not be available, requiring very tight compliance. A possible solution to mitigate such drawbacks could be to use very specific sensors [16,17,18], e.g., electric appliance monitors, pressure pads, and so on. Such sensors have a narrower scope, but allow a more precise and fine-grained targeting of specific actions. This paper presents a cloud-based framework for human behavioral monitoring, exploiting specific smart-home sensors. The proposed system has been successfully deployed at users’ homes within the context of the NOAH [19] and ACTIVAGE [20] projects, funded by the European AAL Joint Programme and Horizon 2020 initiative, respectively. Both projects target older adults, envisaging a continuum-of-care approach. 

We present and evaluate unsupervised methodologies for smart-home data analysis. First, we introduce the general system architecture. Then, behavioral explanatory models are introduced, with the purpose of quantitatively monitoring a given quantity of interest (e.g., toilet visits through time). Results will show that such simple models can be useful as feature extractors for higher-level, more complex models. Then, a tool that models the probability of activation of given sensors throughout the day is presented; this enables a statistical hypothesis testing framework for detecting changes between two different time periods. Finally, we present a methodology to extract complex user patterns by first learning an efficient, compressed representation of collective daily sensor data (leveraging deep learning techniques), and then performing clustering on such a learned space. Results show that behavioral meaningful, multivariate clusters are captured; furthermore, we show that pre-processing data by leveraging deep learning techniques allow obtaining more compact and expressive clusters when compared to unprocessed, standard clustering techniques.

## 2. Materials and Methods

### 2.1. System Architecture and Components

The distributed architecture of the smart home system can be divided into two units: a local, home-based one, and a cloud-based one. In particular, the former continuously collects and streams data, exploiting a standard Wi-Fi connection. The latter, instead, takes care of data ingestion, analysis, and pilot management.

**Figure 1:**
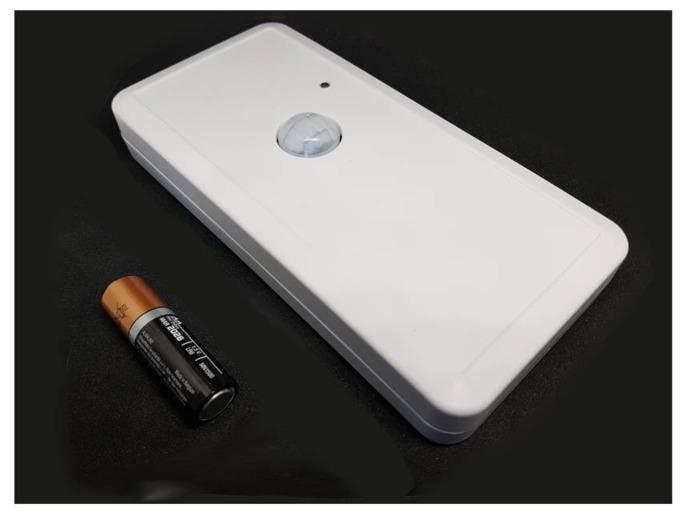
Picture of a fully-assembled Wi-Fi PIR sensor.

At the home level, a Wi-Fi based WSN (wireless sensor network) is created, which may include several of the following sensors (depending on study design and/or pilot environment limitations):

- Passive infrared (PIR) sensors for motion detection, suitable for tracing room occupancy. A picture of a fully-assembled Wi-Fi PIR is reported in Figure 1;
- Magnetic contact sensors, useful for monitoring open/close states of different objects. For example, interactions with doors, drawers and medical cabinets can be easily detected with such sensors;
- Bed occupancy sensor, useful in tracing sleeping patterns;
- Chair occupancy sensor, to gather information on how much time and when a user sits on a chair/armchair/sofa;
- Toilet presence sensor, specifically developed to keep track of daily toilet use;
- Fridge sensor, to detect openings of the fridge door; and
- Power meter, to monitor home appliances use, such as TV, microwave oven, air conditioning, etc.

All sensors are battery-operated (2× AA alkaline batteries) and support secure OTA (over the air) firmware updates, to guarantee easier maintenance. Following a genuine IoT-based approach, sensors are directly connected to the cloud, exploiting the MQTT protocol. Internet connectivity is guaranteed by common Wi-Fi home routers (IEEE 802.15 b/g/n), whereas pairing of such devices with the home network exploits the Wireless Protected Setup (WPS) security standard. In order to guarantee data security, all traffic towards the cloud is encrypted using the standard SSL/TLS protocol. An extra step of protection in terms of data privacy is offered by: (i) restricting sensors to only publish data, preventing retrieval; and (ii) exposing a REST-API interface for the analytics services, protected by secure authentication with username and password. Direct access to pilot data is not possible from the outside; furthermore, user-generated data is pseudo-anonymized, by identifying the patterns with a numeric ID and by only saving user-pilot association in a secure table.

As previously mentioned, the cloud back-end serves many purposes; two high-level tasks can be identified in particular: (i) performing data analytics to get insights from user-generated data, which will be addressed in the Section III; and (ii) managing the operating aspects of the pilots. In particular, the former one can be thought of as a cold path, meaning that it does not operate in real-time on streaming data, whereas the latter can be called a hot path, focusing mostly on the real-time generated data. In the following, we will refer to the hot path as the *streaming analytics pipeline*, whereas the cold path will be referred to as the *analytics pipeline*.

The streaming analytics pipeline must meet several requirements, given our application scenario:

- high-bandwidth handling of incoming messages, originating from multiple IoT devices;
- ability to perform operations on streaming data, possibly being automatically triggered at the arrival of specific messages;
- scalability, allowing future massive deployment of pilots; and
- high service availability, in order to reliably collect and store data.

In order to cope with such tight requirements, we exploit mainstream managed services from major cloud providers, including Microsoft Azure and IBM Bluemix. Those platforms support automatic bandwidth scaling of their services, adjusting the computing capability to meet the actual user demand, by applying predictive load modelling and simple rule-based actions. Similar consideration in terms of scalability and flexibility also apply to the analytics pipeline, which exploits powerful statistical and machine learning techniques to mine and extract information from raw data.

Three main functional units can be identified in our cloud architecture: IoT data ingestion, streaming analytics, and behavior analytics, all detailed below.

*IoT Data ingestion*: high-bandwidth data flow, coming from the home WSN, is ingested exploiting dedicated managed buffering services (Azure IoT Hub/IBM Watson IoT), which can handle multiple, simultaneous IoT MQTT data connections. Data are stored in the buffer for a fixed (configurable) amount of time, allowing worker nodes/functions to efficiently fetch them; such worker nodes are in charge of validating and storing the data into a persistent SQL database.

*Stream Analytics*: the online, real-time processing capability of the system is achieved via dedicated managed services (Azure Stream Analytics/Watson IoT), thus avoiding the need of configuring clusters, and allowing easy scaling capabilities. This part allows efficiently querying of the real-time streaming data and performs simple processing on them, to perform pilot management tasks, such as:

- Detection of the sensor’s low-battery status and triggering of related alarms on the user interface;
- Monitoring sensor connectivity: sensors (even when inactive) send keep-alive messages at a given frequency (e.g., 60 min intervals). Sensor-specific rules can be created to trigger alarms or countermeasures if a fails to communicate; and
- Detection of inconsistent or grossly abnormal values, and triggering of countermeasures, based on simple rules, e.g., a front door kept open for a long period, or prolonged bed occupancy may trigger caregiver notifications.

Users can be notified about important/subscribed notifications through a simple web service.

*Behavior Analytics*: offline data mining and modelling is performed on pilot data saved in the SQL DB. All analytics models are implemented in Python and made available to the user’s requests through the simple REST API.

### 2.2. Data Analysis

In the previous section, we discussed the implementation details of the cloud back-end, introducing two separate paths for sensor data processing. This section focuses on the so-called cold path, i.e., all the analytics are carried out on stored data (i.e., not in real-time), aiming at extracting information and insights on users’ habits. As mentioned in the introduction, analytics are implemented to fulfill three main purposes: (i) to have an objective measure of several quantities of interest (e.g., sleep patterns or daily toilet usage); (ii) to detect changes or trends in said quantities; and (iii) to discover natural habits that the user has. In the following, we will introduce techniques suitable for addressing such requirements. In particular:

- *Behavior explanatory models* (BEM), augmented with *outlier detection* techniques, are useful for precisely assessing the presence of longitudinal trends in a given time series of interest, e.g., nightly bathroom visits. This can be carried out at multiple time resolutions (e.g., *n* weeks, *n* = {2,4,6, …}) and exploiting rolling windows, to detect temporary phenomena.
- *Sensor profiles* (SP) are useful to compare patterns of single sensors from two different periods. Exploiting a statistical hypothesis testing framework, it is possible to objectively assess whether changes in patterns took place or differences between them are just measurement noise.
- *Multivariate habits clusters* (MHC) are useful to find recurrent patterns in user data, possibly involving more sensors simultaneously, and give insights on users’ habits by relaxing the assumption of population comparison as per the direct sensor profile comparison.

#### 2.2.1. Sensor Data Description and Pre-Processing

**Figure 2:**
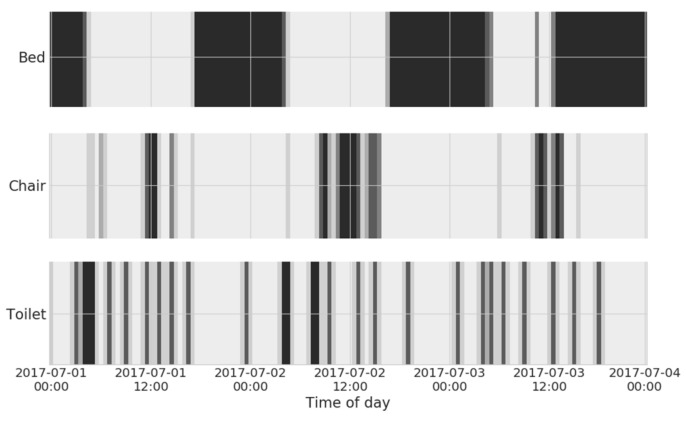
Activation patterns of home sensors, over a three-day timeframe. The darker color represents higher event counts detected within each time bin.

A typical pilot consisting of a bed, chair, door, toilet, and PIR sensors yields an average of approximately 130 events/day, with (firmware-level filtered) PIR sensors accounting for as much as 50% of the total activations. In this regard, sensor-level event filtering plays a major role in power saving: since power drawn from the radio section of the device is the greatest source of energy consumption, it is very important to transmit as little data as possible, ideally filtering all spurious or intermittent activations. In order to further optimize battery life, data are sent in bursts, once per hour. In this setup, it is expected that, on average, a sensor can operate for 6–9 straight months. Raw data produced by the home sensing environment are visualized in the example shown in Figure 2. Of course, such data are not suitable for direct interpretation by the end-user himself or his caregiver: data analytics techniques, introduced below, are, thus, needed to convert such data into useful and accessible knowledge. 

Sensor data are logged in the SQL database according to the flexible schema illustrated in Table 1. The data structure is independent of the actual sensor configurations and allows for easy database querying. In general, not all fields are necessarily exploited, depending on the kind of sensor: for example, the status of binary sensors (e.g., bed, chair, PIR) can be extracted from the *Int_value* field, whereas real-valued sensors (e.g., smart power meters) exploit the *Float_value* field.

The logging scheme is event-based: sensor values are not sampled at a constant frequency, to maximize storage efficiency. However, some analytics approaches may benefit from having regularly-sampled data: for such purposes, we convert event-based data to a resampled, aggregated representation. Typically, we use a 1-min resolution, which is a reasonable trade-off, allowing the capture of fine-grained patterns while preventing excessive memory usage. 

Since all sensors share the same index after resampling, it is possible to represent the status of the smart home in matrix form: each column represents a given sensor, whereas rows represent the resampled time intervals. We call such entities *signature matrices*. This approach works for both discrete and real-valued sensors; nonetheless, in order to extract activation patterns, it is always possible to discretize a real-valued sensor (e.g., TV power consumption meter) by applying a suitable threshold filter. If all sensors carry just binary information, we have a binary signature matrix.

#### 2.2.2. Behavior Explanatory Models (BEM)

Once resampled, basic statistics and activation patterns may be derived: such information is presented to the users of the analytics system as daily sensor dashboards, detailing all activations (time active and distinct event counts). A deeper analysis level is provided by the use of interpretable generalized linear models (GLM) [21]: they allow quantifying the presence of linear trends and many other (possibly periodic) factors, such as weekend days. Our cloud-based analytics engine supports automated regression analyses on each sensor data stream. Essentially, two main analysis types are adopted, depending on the nature of the sensor and/or the quantity of interest:

- *Real-valued* regressions. This operation may be conducted by exploiting different frameworks, such as ordinary least squares (OLS), ridge regression, lasso, Bayesian regression, and many others. This kind includes, for example, the amount of time a person spends sitting, lying in bed, watching TV, and so on.
- *Discrete-valued* regressions. This kind includes, for example, the number of toilet visits during night or the whole day, the number of awakenings from bed, and so on. These quantities are better modeled as discrete random variables, therefore suggesting the application of Poisson/negative binomial regression frameworks.

It is worth noting that a given sensor is not bound to a linear or Poisson model exclusively, but rather it may contain relevant information for both classes. For example, a bed sensor can be used to monitor both the amount of hours spent in bed (continuous value), as well as the number of awakenings during the night (discrete value). All regression models are applied on a given time-window, or are repeatedly applied to different intervals. Furthermore, such models can be applied in a rolling-window fashion, thus allowing the discovery of temporary trends. 

The purpose of such regressions is to yield interpretable results, allowing the assessment of the impact that different factors have on the observed data. Common features that we include in those regressions are, for instance:

- *bias term*: the average event count or measure;
- *linear trend term*: a linearly-varying feature trying to detect longitudinal increments (or decreases); and
- *weekend day*: a binary feature, which is false during weekdays and true for weekends. This allows capturing differences between those two categories.

This analysis is then augmented with outlier detection. It is worth remarking that, given the nature of the experimentation, which does not consider active tagging and annotation by end users, all the analyses are run in an unsupervised way. In general, first gross outliers are removed by basic pre-processing (e.g., based on the inter-quartile range and dropping of null values). Then, a pool of models is fitted to the observed data, exploiting different combinations of the abovementioned features. Model selection is then performed, based on the Akaike information criterion (*AIC*):

(1) AIC=2k−2ln L,

where *k* is the number of parameters (features) of the model, and *L = P(data|θ,M*) is the likelihood of the data given model *M* and best fitted parameters *θ*. The model with the lowest AIC score is selected, being the one that best fits the data while being least complex (i.e., with as few factors involved as possible). Once fitted, regression models can also be useful to spot outliers in the data.

**Figure 3:**
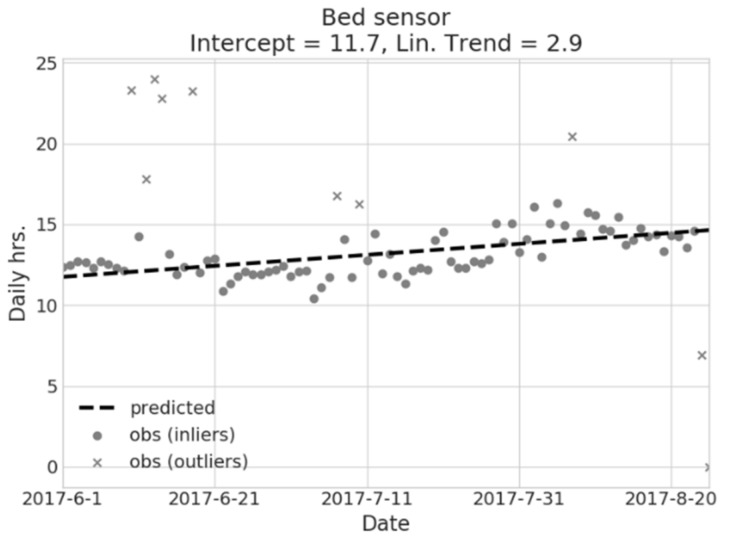
Example of the BEM model from a GLM regression on the bed usage hours during the night. Usage prediction is shown, as are detected anomalies (outliers).

For instance, Figure 3 illustrates the application of regression model to bed occupancy time: the figure refers to a real case of a 78 year old male subject, living alone. It is shown that an average of about 11.7 h is spent in bed during the observed period, and a significant increase with time is evident (≈+2.9 h, over the observed period). Given such a figure, this behavior can only be partially ascribed to the seasonal, increasingly hot climate, and can, thus, be reported to the caregiver, triggering further investigation. It is important to note that, given the explanatory purpose of the model, such a trend has not been interpreted as a prediction for subsequent periods, but rather as a significant attribute of underlying data. The model also detects the presence of outliers (grey crosses in the scatter plot). Furthermore, feature weights and outlier information can be used to feed higher-level models to perform deeper behavioral inference, as mentioned in the Results section.

#### 2.2.3. Sensor Profile (SP) Comparison

Sensor profiles (SP) attempt to model the expected activation profile of a sensor as a function of the time of the day. Once again, depending on the nature of the data (discrete or real-valued), different procedures are used to compute such profiles:

- For real-valued data, we estimate the expected percentage of time a sensor is seen as active within a given time bin; and
- For discrete-valued data, the expected probability of seeing an activation within a given time bin is estimated, instead. This is particularly useful for more “impulsive” (i.e., sparsely-activated) data, such as fridge/drawer opening or toilet visits.

**Figure 4:**
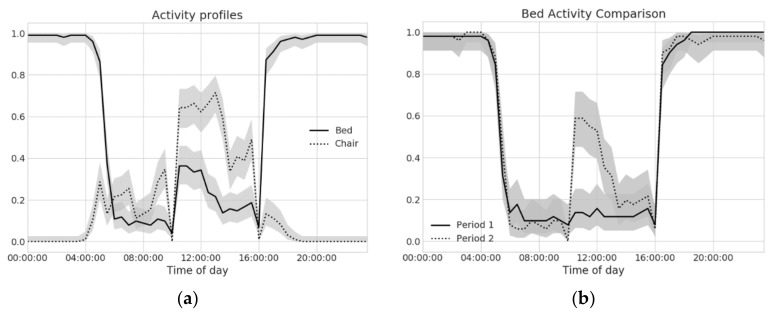
(**a**) Sensor profiles for bed and chair sensors. The shaded area represents the 95% confidence interval of the estimated sensor profile values; and (**b**) a comparison of bed SP for two consecutive periods (20 days each). Non-overlapping confidence intervals indicate statistically significant differences, indicating underlying behavioral changes. In this case, bed presence is significantly increased right after lunch (time in UTC).

The choice of the bin size plays an important role: setting bins that are too long may lead to a suppression of relevant behavioral information, whereas bins that are too short may yield noisy estimates. For each bin, a bootstrapping procedure is used to derive the distribution estimates and confidence intervals from daily signature matrices. Figure 4a shows the activity profiles, at 30 min time bin resolution, for bed (solid line) and chair (dotted line) sensors, along with their confidence intervals (shaded areas around each curve).

Sensor profiles also offer a basis to perform behavior change detection. In this scenario, two different time periods (e.g., the last three weeks against reference ones) are compared. For each time bin, the populations (daily signature matrices data) of the two populations are statistically compared exploiting a hypothesis testing framework (e.g., parametric or permutation tests, if the sample size is limited). This procedure is repeated for each time bin of a full day, and the resulting *p*-values are adjusted using the Holm-Bonferroni procedure, in order to account for multiple comparisons. For example, Figure 4b compares bed sensor profiles for two consecutive periods, each spanning over 20 days. A comparison of the profiles allows detecting a statistically significant change of behavior (*p* < 0.01) between 10:30 a.m. and 12:30 p.m. (UTC time, local time is UTC + 2 h), which is made evident by non-overlapping confidence intervals. In particular, an increased activity in the second period (dotted line) is noticeable, with respect to the preceding one (solid line). Such an increase is consistent with the regression plot in Figure 3, with the profile analysis providing the caregiver with more detailed and expressive description.

#### 2.2.4. Multivariate Habits Clustering (MHC)

Explanatory models and sensor profile are good tools to monitor and compare a given quantity across time (days for BEM, daily time bins for SP). Such extracted information can be used to trigger automatic notifications to caregivers of underlying behavioral changes. However, both BEM and SP do not provide information about general user habits: they are mainly univariate analysis tools. General user habits, instead, are better analyzed as multivariate quantities, considering interactions between different sensors. This can be accomplished by suitable data fusion techniques. In the following, we present a deep-learning based method to cluster the multi-variate signature matrices data, leading to the extraction of representative user patterns.

Recent advances and breakthroughs in *Artificial Neural Networks* (ANN) suggest that Deep Learning techniques can be leveraged to automatically extract high-level features from data. An interesting application of Deep Learning is unsupervised representation learning: the ANN learns a suitable embedding, with which data can be represented in a potentially lower dimension, while preserving all relevant characteristic. In particular, *AutoEncoders* (AE) [22] offer a good framework to implement such representation learning. Input data to an AE is first transformed to a hidden dimension by means of an encoder unit; such inner representation is then remapped back to the original input space by means of a decoder unit. The AE is trained by forcing the output to be as close as possible to the input: a loss function is applied to quantify such reconstruction error between input and output, and this information is used to adjust both encoder and decoder parameters to minimize it. Typical loss function choices are the well-known mean squared error or, for binary outputs, log loss:

(2) $$L\left(w\right)=-\frac{1}{N}\overset{N}{\underset{n=1}{\sum }}\left[y_{n}\log{\hat{y}}_{n}+\left(1-y_{n}\right)\log\left(1-{\hat{y}}_{n}\right)\right],$$

where 0 < *y* < 1 is the true output and *ŷ* the predicted one and ***w*** the parameters of the net. Learning is then typically accomplished by means of *Stochastic Gradient Descent* (SGD) or its derivations, such as *rmsprop* or *adam* [23]. If learning converges to a small reconstruction error, the inner representation provided by the encoder output can be used to represent data into a new lower dimensional space. AE can be trained separately and then stacked on top of each other to achieve higher-level representation (*Stacked-AE*, SAE).

Our case involves multivariate time series data, consisting of sensors signature matrices. Two main frameworks are used to deal with such data:

- *Recurrent Neural Networks* (RNN), among which LSTM (*Long-Short Term Memory*) [24] and GRU (*Gated Recurrent Units*) [25] have proven great potential in extracting relevant features, being capable of capturing both fine grained and long term time dependencies in the data. They are commonly exploited in sequence to sequence prediction problems, including machine translation and time series forecasting. A GRU cell can be described as being composed of a memory cell, an update gate and a reset gate. The cell is responsible for remembering values over arbitrary time intervals, whereas the gates control the flow of information from input data, previous output data and the cell state itself. Mathematically, a GRU can be described by the following equations:
  (3) $$\{\begin{array}{c} z_{t}=\sigma_{g}\left(W_{z}x_{t}+U_{z}h_{t-1}+b_{z}\right)\\ r_{t}=\sigma_{g}\left(W_{r}x_{t}+U_{r}h_{t-1}+b_{r}\right)\\ h_{t}=\left(1-z_{t}\right){}^{\circ}h_{t-1}+z_{t}{}^{\circ}\sigma_{h}\left[W_{h}x_{t}+U_{h}\left(r_{t}{}^{\circ}h_{t-1}\right)+b_{h}\right] \end{array},$$
  where the operator ° denotes the Hadamard product (entry-wise product), subscript *t* and *t* − 1 refer to current and previous time-step, respectively, *x_t_* is the input vector to the GRU unit, *z_t_* the update gate’s vector, *r_t_* the reset gate’s vector, *h_t_* the output vector of the GRU unit, *σ_g_* is the sigmoid activation function, *σ_h_* is the hyperbolic tangent activation function, and matrices *W_k_*, *U_k_*, *b_k_*, *k = {z, r, h}* are weight and bias terms that need to be learned from data.
- *1D Convolutional Neural Network* (CNN). CNN have largely become popular in machine vision tasks for their ability to find scale-invariant features, when coupled with max-pooling techniques. By performing convolutions only along the temporal axis (Conv1D nets) [26], it is possible to extract relevant features for time series prediction. In general, a convolutional layer operates as follows:
  (4) $$y^{i}=f\left(b^{j}+\underset{i}{\sum }k^{ij}*x^{i}\right),$$
  where *x_i_* and *y_j_* are the *i*-th/*j*-th input/output map, *k_ij_* is the convolution kernel (whose size is also called the receptive field) between the *i*-th input map and the *j*-th output map, * is the convolution operator, and *f* the activation function. In our case, activation is *ReLU* [27]:
  (5) ReLU(x)=max({0,x}).
  *Max pooling* is defined as taking the maximum value over a given receptive field, for example a *kxk* patch in 2D or a *kx*1 patch in 1D. Each convolutional layer is characterized by a parameter, i.e., the number of filters, which expresses how many features representations will be learned by it.

**Figure 5:**
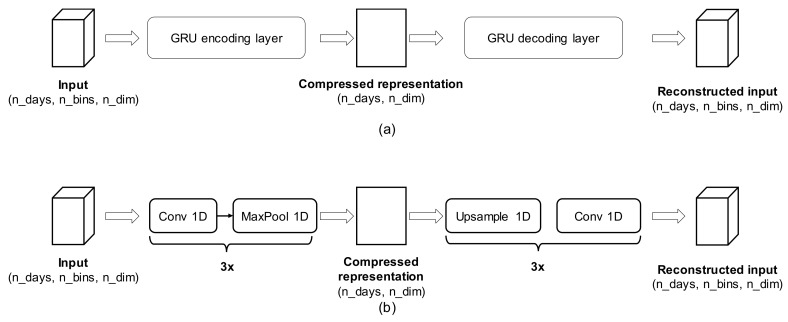
AutoEncoder (AE) architectures for learning the compressed representation of daily multi-sensor profiles. (**a**) GRU (gated recurrent unit) based AE; and (**b**) one-dimensional convolutional (CONV1D) AE.

We define two AE architectures, based on LSTM and Conv1D, to learn a compressed representation of input data, as shown in Figure 5. In particular, the AE is forced to adjust encoder and decoder parameters so that the log-loss in Equation (2) is minimized. Once trained, the representation learned by the encoder part of the AE is used as input feature for standard clustering algorithms, such as *k*-means, Spectral Clustering Mean-Shift. As a result, points that are close in the learned embedding, will be clustered together. Once the clusters are determined, we can extract their prototypes, defined as mean or median aggregates, in the original sensor data space, of samples belonging to the same cluster.

## 3. Results

We tested the methodologies introduced above on real-life data, coming from multiple smart homes. The target population consists of elderly individuals, 70 years or older, living alone (or having a private room in caregiving homes) with no major clinical conditions. All methods introduced in the previous section are exposed as RESTful web services: results are mostly computed in near real-time, with the exception of sensor cluster extraction, which are computed on a daily basis, given the relatively high computational cost (which would cause lags too long to be perceived as real-time).

### 3.1. Behavioral Explanatory Models

Behavioral explanatory models were tested on available real data. After the confirmation of detected trends and outliers by visual inspection, we stressed the system by artificially altering (increasing, decreasing, removing) trends. In all cases, the BEM models were able to correctly adjust the estimate. Furthermore, the AIC criterion ensured that models with unnecessary factors were not selected (e.g., weekend-day feature).

As mentioned before, results from basic analytics models can be used as input features for higher-level models. We tested if the coefficients extracted by BEM may be useful to discriminate patterns of different users. Such coefficients were extracted on a daily basis (rolling window approach) using each time data of the last 15 days. In particular, we ran daily BEM on the following quantities:

- Overall bed presence (real-valued, unit: hours);
- Nightly (7 p.m.–9 a.m.) bed presence (real-valued, unit: hours);
- Overall chair presence (real-valued, unit: hours);
- Electric appliance use (real-valued, unit: hours);
- Toilet sensor (distinct event counts); and
- Door sensor (distinct event counts).

**Figure 6:**
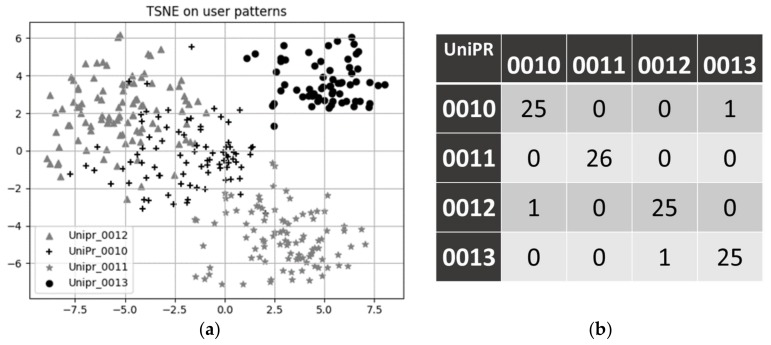
(**a**) 2-D visualization of daily living patterns as projected by the *t-SNE* manifold-learning algorithm; and (**b**) the confusion matrix, on the test set, for the user classification problem.

Coefficients extracted by BEM may then be combined, on a daily basis, and used as features to describe each users’ day. In the following, we show an example of such use; in order prevent cluttering, we restrict the analysis to about three months of data from four different pilots (labelled *UniPr_00XX*, with *XX* = {10, 11, 12, 13}). A nice 2D visualization of such extracted features is shown in Figure 6a as a result of a projection using the *t-SNE* algorithm [28]. It appears that the *t-SNE* algorithm is able to learn an efficient embedding of the data in terms of user clusters: patterns belonging to the same user are, indeed, mapped close to each other. Furthermore, it can be observed that data generated from pilot *UniPr_0013* (solid black dots) appears to be relatively compact and somehow far from the remaining ones. If we consider that such a pilot is actually located in a structure different from the retirement home where other users (pilots *UniPr_0010*, *UniPr_0011*, *UniPr_0012*) live, this suggests the method may actually recognize lifestyle patterns in an effective fashion. We also investigated the discriminating power of the extracted features, by addressing the following classification problem: given the extracted features, what is the pilot that most likely generated them? More specifically, we set up a SVM (support vector machine) classifier, and assessed its performance on user recognition. An average *f1* score of 0.97 on the test set was achieved, indicating a good classifier skill. The associated confusion matrix is reported in Figure 6b, for completeness’ sake. Of course, this experiment just aims to demonstrate the possibility of learning a multi-variate description of a user’s day. In the given example, we show an expressive comparison among different users. By accumulating more data, the same approach can be applied to just a single-user dataset; in this case, a multivariate model about daily feature distributions may be derived and used to perform anomaly detection by standard techniques, such as one-class SVM [29] or isolation forest [30].

### 3.2. Sensor Profile Comparison and Multivariate Habit Clustering

SP were computed on real pilot data, and comparisons were carried out. An example of the successful detection of a behavioral change was provided earlier in Figure 4b, where bed presence increased around early afternoon during the second period. This was also confirmed by a linear trend in overall bed presence, spotted by BEM models. We also introduced a perturbative approach, to investigate how the system reacts to temporary increases in a given sensor use around a specific time slot. What emerged is that the choice of the length of the periods to be compared impacts the sensitivity of the algorithm. In particular, longer periods (larger populations) allows detecting smaller changes; however, on the other hand, periods that are too long may over-smooth the data, possibly missing temporary changes. We suppose that a multi-resolution approach may help mitigate this issue, and this will be further addressed in future work.

**Figure 7:**
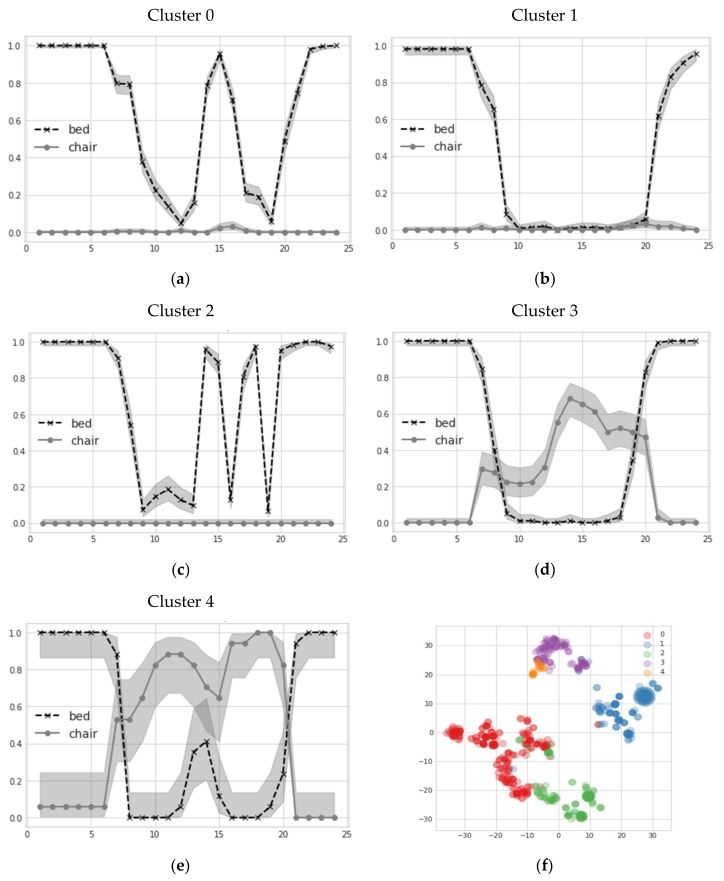
(**a**–**e**) Examples of MHC clusters extracted by GRU-based embedding; (**f**) 2D visualization (obtained by applying the *t-SNE* manifold learning algorithm) of embedded space induced by GRU. Different colors refer to the clusters found in the sub-figures (**a**–**e**).

As previously stated, SP are useful analytic tools to compare precisely-defined quantities of interest (e.g., daily sleep patterns). With the availability of a large number of daily records, we also investigated the multi-variate aspects of spontaneous user habits, exploiting the previously-mentioned MHC method. As previously mentioned, two autoencoders were trained, based on GRU cells and Conv-1D units (Figure 5a,b, respectively), to self-learn an efficient embedding of daily sensors profiles. It is important to stress that such embedding, automatically learned from the data, allows combining multiple sensors at once, generalizing the vision offered by single sensor profiles. Such embeddings are then used to map daily multi-variate patterns to a compact representation, over which standard mean-shift clustering is performed. Results of emerged clusters for GRU-based AE are reported in Figure 7a–e, considering bed and chair sensors simultaneously. Clear behavioral patterns emerge, interpretable as multi-sensor profiles. It can be noticed that clusters 0 and 2 are quite similar, having the user rest on bed during the afternoon. Clusters 1 and 3 have similar bed patterns, but differ for chair utilization during the morning and afternoon. Finally, cluster 4 is similar to 3 in chair use through the morning and afternoon, but puts some small probability of finding the user in bed just around noon. Such similarities are more explicit if we plot a 2D representation of the learned embedding by exploiting *t-SNE* dimensionality reduction. From Figure 7f we observe that similar clusters tend to be represented in neighboring regions of the 2D space. Similar results, with similar extracted clusters, apply for the Conv-1D AE. 

For comparison, we also directly clustered sensor profiles by concatenating different sensors belonging to the same date. The same clustering algorithm is applied, exploiting the same Euclidean distance norm. Interestingly, the number of clusters extracted using this technique is far greater than those found by AE pre-processed ones: the first five clusters extracted via Conv-1D and GRU AE account for about 95% of the data, whereas this percentage drops to 78% for regular clustering. For completeness, we report the silhouette score to compare the compactness of extracted clusters. In particular, we found GRU-based clustering resulted in the highest silhouette score (0.34), followed by Conv-1D (0.31) and regular clustering (0.23).

## 4. Discussion

Daily life monitoring may help in preserving quality of life of older adults, allowing them to maintain their independence and live longer and safer in their own home. An approach based on indirect, non-intrusive monitoring may effectively complement more conventional telemedicine techniques. To this purpose, a system based on a simple wireless sensor network, suitable for home use, has been designed, implemented, and tested. Machine-learning techniques are exploited to infer meaningful information from sensor data, dealing with the inherent variability of human behaviors and avoiding user-dependent system calibration. In order to implement such advanced analytics services, the system relies on a cloud-based architecture, which avoids the need of maintaining complex and expensive data-processing architectures at home. This allows for lower costs, high scalability, and interoperability. 

The system has been deployed at several pilot sites, in the framework of the NOAH AAL-JP and ACTIVAGE H2020 projects. Overall, pilot sites are located in different European countries, aiming at assessing its performance in a real-life environment while accounting for social, economic, and cultural differences across Europe. At the time of writing, about 20 full pilot kits were deployed, and over 300,000 pieces of event data were collected, with no major issues in local and cloud operation; about 80 further pilots are foreseen in the near future.

Overall pilot size and duration were meant for technical validation and for implementing user-centered design iterations: a wide range of stakeholders were involved in such phases, including older adults, their relatives, formal and informal caregivers, and public and private entities dealing with home-care services.

Larger-scale testing will be needed to assess the effectiveness and impact on care practices: in this paper, we have focused mostly on discussing the feasibility and practicality of continuous, indirect monitoring strategies. Much effort was devoted to extracting simple, usable, and reliable information from sensor data, relieving the end-user or the caregiver from interpretation tasks. A modular set of analytical functions was developed and implemented. Such functions, featuring a fully unsupervised, inherently adaptive approach, resulted in the automatic detection of relevant trends and anomalies and providing useful building blocks to be integrated into caregiving services. 

Of course, the system does not claim to provide actual “diagnostic” capability, which is still the competence and responsibility of health professionals. Instead, it may act as a “magnifying glass”, capable of making more evident behavioral insights, which could possibly be relevant, but are likely not to be noticed otherwise. 

The test in pilot environments provided a convincing proof-of-concept test, demonstrating the ability of eliciting expressive indications from indirect, continuous monitoring in a fully automatic fashion. 

The approach could, therefore, effectively integrate current caregiving practices, aiming at a multidimensional, more continuous assessment of older adults’ wellbeing and health. This will foster their independent life and support the quest for sustainable solutions to the demographic challenges.

## Figures and Tables

**Table 1:** SQL database organization.

Field	Description
**Gtw_id**	Gateway unique identifier
**Obj_id**	Sensor unique identifier (within the same Gtw_id domain)
**Variable_id**	Identifier of the variable (e.g., sensor status, battery level etc.)
**Int_value**	Value of the variable, integer
**Float_value**	Value of the variable, floating point
**String_value**	Value of the variable, string
**Flags**	Internal use
